# A novel series of high-efficiency vectors for TA cloning and blunt-end cloning of PCR products

**DOI:** 10.1038/s41598-019-42868-6

**Published:** 2019-04-23

**Authors:** Ken Motohashi

**Affiliations:** 1 Department of Frontier Life Sciences, Faculty of Life Sciences, Kyoto Sangyo University, Kamigamo Motoyama, Kita-ku, Kyoto 603-8555 Japan; 2 Center for Ecological Evolutionary Developmental Biology, Kyoto Sangyo University, Kamigamo Motoyama, Kita-Ku, Kyoto 603-8555 Japan

**Keywords:** Genetic engineering, PCR-based techniques

## Abstract

An efficient PCR cloning method is indispensable in modern molecular biology, as it can greatly improve the efficiency of DNA cloning processes. Here, I describe the development of three vectors for TA cloning and blunt-end cloning. Specifically, pCRT and pCRZeroT were designed to improve the efficiency of TA cloning. pCRZeroT can also be used with pCRZero to facilitate blunt-end cloning using the *ccdB* gene. Using pCRZero and pCRZeroT and applying the Golden Gate reaction, I developed a direct PCR cloning protocol with non-digested circular vectors and PCR products. This direct PCR cloning protocol yielded colony-formation rates and cloning efficiencies that are comparable with those obtained by conventional PCR cloning with pre-digested vectors and PCR products. The three plasmids I designed are available from Addgene (https://www.addgene.org/).

## Introduction

Polymerase chain reaction (PCR) is an indispensable tool for amplification of genomic DNA and transcripts to analyze their functions^1^. Improvements to the basic technique to facilitate highly efficient molecular cloning of PCR products into a vector are required to promote research projects. Various types of thermostable DNA polymerases are commercially available for PCR amplification. Of these, Taq DNA polymerase is the most widely used; this enzyme attaches a deoxyadenosine triphosphate (dA) to the 3′-end of amplified DNA^2^. In contrast, various high-fidelity thermostable DNA polymerases that possess 3′ to 5′ exonuclease activity for proofreading, such as Pfu DNA polymerase^3^, KOD DNA polymerase^4^ and Phusion DNA polymerase^5^, produce the blunt-end DNA fragments. Due to the diverse types of PCR product that are generated, choice of cloning vector is a critical step that can ultimately determine cloning efficiency^6^.

Vectors that have a T-overhang at the 3′-end were developed as so-called ‘T-vectors’ for cloning of dA-tailed PCR products amplified by Taq DNA polymerase^7,8^. To prepare the T-overhang vector from general cloning vectors, dideoxythimidine triphosphate (ddT) is incorporated at the 3′-terminus of a linearized blunt end vector by using terminal deoxynucleotidyl-transferase^7^. Unfortunately, this strategy is not widely adopted by researchers, as the reagents are not frequently used. The availability of commercially available ready-to-use T-vectors has obviated the need for very specific and complicated procedures; however, while the vectors are generally very convenient, they are expensive. Another T-vector preparation-method uses XcmI (a well-known type IIS restriction enzyme that recognizes an asymmetric nucleotide sequence) to digest outside of this recognition sequence and generate a T-overhang for TA cloning^8^. To use these T-vectors, the two XcmI sites that are designed to produce the 3′-end T-overhang are introduced into a specific vector^9–11^. Preparation of T-vectors by XcmI cleavage is a relatively easy way to produce T-overhangs, because XcmI is a commonly used restriction enzyme. However, XcmI-dependent T-vector preparation is hampered by a high background colony rate; this is attributable to re-ligation of small excised DNA fragments into the digested empty vector^9,10^.

In contrast to TA cloning using a single nucleotide T-overhang, blunt-end cloning of PCR products is less efficient because the terminal ends of the DNA fragment are not sticky^12,13^. To overcome the low efficiency, ZERO-background-cloning using the *ccdB* gene, an active cytotoxic factor in *Escherichia coli*, was developed as a positive-selection system^14^. *ccdB*-sensitive *E. coli* cells carrying empty vectors cannot survive in this system because the expression of CcdB protein is lethal^15^.

Based on their needs, researchers should therefore choose either a T-overhang vector or a blunt-end vector including the *ccdB* gene^14,16,17^. Ideally, a complete vector system that covers both choices should be available. However, such a vector has been realized only partially. pPCV can be used for both TA-cloning and blunt-end cloning, but is not applicable to positive screening because the plasmid does not contain the *E. coli*-lethal *ccdB* gene^13^. pBS2ndd and pBS3ndd are also utilized as bifunctional vectors for TA-cloning and blunt-end cloning, but screening of the vectors requires blue/white selection using the *lac*Zα gene^18^. pCAMBIA1300^19^ and modified pGreen vectors^20^, which contain the *ccdB* gene, can be applied to the ZERO-background screening system of TA-cloning for plant functional genomics, but not to blunt-end cloning. Attempts have also been made to develop bifunctional pKILPCR vectors using the ZERO-background screening system for both TA-cloning and blunt-end cloning^21^. However, the linearization of T-overhang of pKILPCRs by type IIS restriction enzyme AspEI (Eam1105, AhdI) lacked adequate reproducibility and the vector showed low cloning efficiency owing to the contaminant exonuclease activity in the AspEI, which led to the blunting of DNA fragments.

In this study, I developed a series of three types of PCR cloning vectors to meet the need for a vector suitable for both TA and blunt-end cloning. Further, I demonstrate here the utility of this vector series using a direct PCR cloning protocol. This protocol inserts PCR products into non-digested circular vectors via the Golden Gate reaction that can simultaneously digest, ligate, and assemble several DNA-fragments into a vector^22,23^. All three PCR cloning vectors (pCRT, pCRZero, and pCRZeroT) are available to researchers via the Addgene repository (https://www.addgene.org/).

## Results and Discussion

### Design of plasmid vectors for PCR cloning

**Figure 1:**
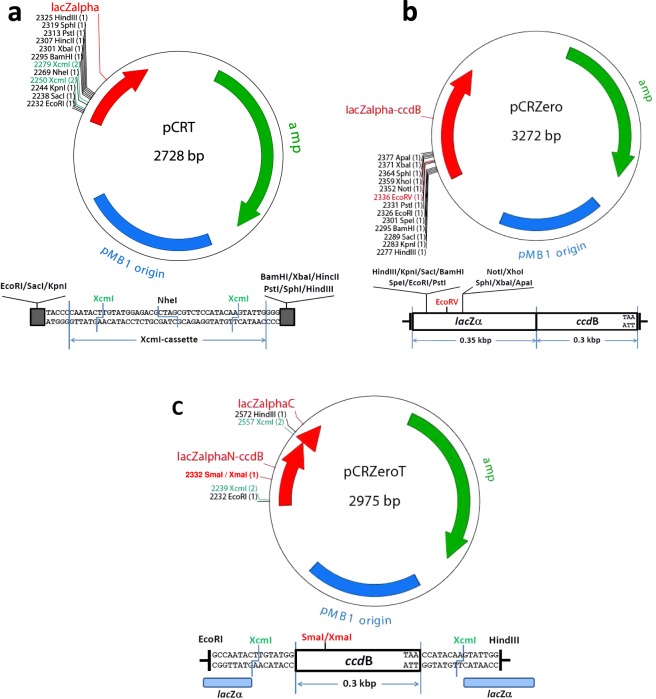
Graphical maps and cloning site regions of pCRT, pCRZero and pCRZeroT plasmids. The restriction sites for TA cloning are indicated with green letters, and the restriction sites for blunt-end cloning are indicated with red letters. The maps were drawn by ApE v2.0.55 software. (**a**) Graphical map and cloning site regions of pCRT. The pCRT was digested by XcmI for TA cloning. (**b**) Graphical map and cloning site region of pCRZero. The pCRZero was digested by EcoRV for blunt-end cloning. (**c**) Graphical map and cloning site region of pCRZeroT. pCRZeroT was digested by XcmI for TA cloning, or by SmaI for blunt-end cloning. A SmaI site in the *ccdB* gene of pCRZeroT was introduced without altering the CcdB amino acid sequence using the SLiP method^35^; this site was used as the cloning site for blunt-end PCR products.

I constructed three plasmid vectors for PCR cloning in this study (Fig. 1). In pCRT (2,728 bp) for TA cloning, T-overhang vector for cloning of dA-tailed PCR products amplified by Taq DNA polymerase can be prepared with one step digestion by a type IIS restriction enzyme, XcmI (Fig. 1a). An NheI site was also introduced into the pCRT vector to reduce background colony formations due to empty vector. In pCRZero (3,272 bp) that is used for blunt-end cloning, positive colonies are selected when the *lacZα*-*ccdB* fusion gene in the vector is disrupted due to insertion of PCR products at an EcoRV site in the vector (Fig. 1b). The inserted PCR fragments can be excised from pCRZero with twelve restriction enzymes (Fig. 1b). The pCRZeroT (2,975 bp) vector is compatible with both TA cloning and blunt-end cloning (Fig. 1c). The SmaI site on the *ccdB* gene in pCRZeroT was introduced as the cloning site for positive selection of blunt-end cloning. pCRZeroT can be also prepared as a T-vector for TA cloning by performing a one-step digestion with XcmI. pCRZeroT has an additional SmaI/XmaI restriction site between two XcmI restriction sites; this reduces background colony-formation due to empty vectors that persist after TA cloning. The introduced SmaI site (CCC|GGG) in pCRZeroT can also be used as an XmaI site (C|CCGGG) to reduce background colony formation (Fig. 1c). The DNA sequence and the plasmid text map of three plasmids are attached in Supplementary Dataset S1. Primers listed in Table S3 can be used for DNA sequencing of cloned PCR-fragments (Table S3).

### Lethality of the CcdB protein in *E. coli* cells carrying *ccdB*-encoding plasmids

**Figure 2:**
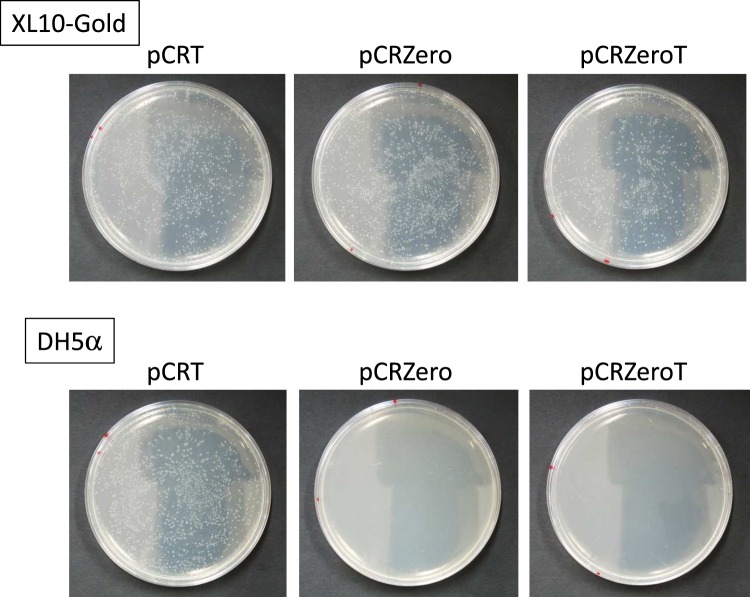
Lethality of the plasmids carrying the *ccdB* gene in the *ccdB*-sensitive *E. coli* strain. *E. coli* XL10-Gold (the *ccdB*-resistant strain) and DH5α (the *ccdB*-sensitive strain) were transformed with pCRT, pCRZero and pCRZeroT plasmids (0.1 ng). The transformation efficiency of the *E. coli* chemically competent cells (XL10-Gold and DH5α) was ~1 × 10^7^ CFUs/μg of pUC19 DNA.

Expression of the *ccdB* gene is lethal in *E. coli ccdB*-sensitive strains, such as DH5α^15,24^. PCR cloning using the pCRZero and pCRZeroT plasmids, which encode the *ccdB* gene, reduce empty vector-dependent background because *E. coli ccdB*-sensitive strains carrying empty vectors cannot form colonies following plating. *E. coli* lethality caused by expression of the *ccdB* gene was checked by transformation of the *ccdB*-sensitive strain, DH5α, with pCRZero and pCRZeroT (Fig. 2). The *E. coli* strain XL10-Gold (a *ccdB*-resistant strain) carrying pCRT, pCRZero and pCRZeroT efficiently formed colonies at the expected frequency (Fig. 2, XL10-Gold). In contrast, no colonies were formed by DH5α (the *ccdB*-sensitive strain) carrying pCRZero and pCRZeroT (Fig. 2, DH5α). On the other hand, DH5α carrying pCRT formed approximately the same number of colonies as XL10-Gold carrying pCRT. These results indicate that pCRZero can reduce empty vector-dependent background during blunt-end cloning, and that pCRZeroT can also reduce the background during both TA cloning and blunt-end cloning.

### Evaluation of cloning efficiencies for TA cloning and blunt-end cloning with the novel vector series

The cloning efficiencies of pCRT and pCRZeroT during PCR cloning were evaluated by TA cloning of dA-tailed PCR products. Both pCRT and pCRZeroT vectors could be screened using the blue/white selection system based on lacZα complementation (Table 1)^25^. A pair of T-overhangs can be prepared with single digestion at XcmI restriction sites in pCRT and pCRZeroT (Fig. 1a,c). However, excised fragments from the vector region can be re-ligated to XcmI sites in pCRT or pCRZeroT. In fact, pCRT and pCRZeroT prepared by digestion with a single restriction enzyme, XcmI, exhibited a ratio of white colonies to total colonies of 38.0 ± 2.4% (for pCRT (XcmI digestion)) and 96.3 ± 6.5% (for pCRZeroT (XcmI digestion)) (Table 1). To avoid the possibility that excised fragments were re-ligated to the vectors, NheI or XmaI sites were introduced into pCRT or pCRZeroT, respectively (Fig. 1a,c). In double digestion (XcmI/NheI for pCRT and XcmI/XmaI for pCRZeroT), white colony formation ratios were improved to 57.0 ± 6.8% (for pCRT) and 100.0 ± 0.0% (for pCRZeroT) when compared to single digestion by XcmI (Table 1). In contrast, the PCR cloning efficiencies were similar in the context of both single and double digestions. As a control for commercially available T-vector, pGEM-T Easy was used for TA-cloning (Table 1). Ratio of white colonies using pGEM-T Easy was 11.9 ± 0.5% and lower than the ratio of white colonies using pCRT and pCRZeroT, although the cloning efficiency was higher than that of pCRT and pCRZeroT. These results showed that the T-vectors developed in this study had higher cloning efficiency than the commercially available pGEM-T Easy.

**Table 1:** Efficiencies with which dA-tailed PCR products were cloned into the T-vectors.

Vector (Restriction enzymes for linearization)	White colonies (%)^*a^	Cloning efficiencies^*b^
pCRT (XcmI)	38.0 ± 2.4	8/16
pCRT (XcmI/NheI)	57.0 ± 6.8	8/16
pCRZeroT (XcmI)	96.3 ± 6.5	7/16
pCRZeroT (Xcm/XmaI)	100.0 ± 0.0	6/16
pGEM-T Easy	11.9 ± 0.5	15/16

T-overhangs were generated in pCRT and pCRZeroT with the indicated restriction enzymes before the ligation reaction. The T-vectors and dA-tailed PCR products (G6PDH1; 1.6 kbp) were purified using a Gel/PCR Extraction Kit. The T-vectors (50 ng) and the dA-tailed PCR products (G6PDH1, 130 ng) were ligated using the ligation convenience kit (Nippon Gene). Half the volume of the ligation mixture was used to transform 50 μL of *ECOS* Competent *E. coli* DH5α chemically competent cells. Commercially available T-vector pGEM-T Easy was used as a control.

^*a^The fraction of white colonies as a percentage of total colonies was calculated and expressed as mean percentage ± standard deviation of three independent experiments.

^*b^The cloning efficiencies are represented as “the number of clones with the confirmed correct length of insert DNA by colony PCR/number of white colonies subjected to colony PCR”.

The PCR cloning efficiencies of pCRZero and pCRZeroT were evaluated by blunt-end cloning of PCR products amplified with Tks Glex DNA polymerase. Both pCRZero and pCRZeroT vectors could be screened by a ZERO-background cloning system using the *ccdB* gene (Table 2). In fact, both blunt-end ligations of pCRZero (EcoRV) and pCRZeroT (SmaI) yielded high cloning efficiencies in blunt-end PCR cloning of PrxIIE (0.6 kbp) and G6PDH1 (1.6 kbp) (Table 2).

**Table 2:** Efficiencies with which blunt-end PCR products were cloned into the blunt-end vectors carrying the *E. coli*-lethal *ccdB* gene.

Vector (Restriction enzyme for linearization)	Insert	Cloning efficiencies^*a^
pCRZero (EcoRV)	Prx IIE	21/24
pCRZero (EcoRV)	G6PDH1	22/24
pCRZeroT (SmaI)	G6PDH1	23/24

The pCRZero and pCRZeroT were digested with indicated restriction enzymes. The digested blunt-end vectors and, blunt-end PCR products (Prx IIE (0.6 kbp) or G6PDH1 (1.6 kbp) were purified by a Gel/PCR Extraction Kit. The blunt-end vectors (50 ng) and the blunt-end PCR products (Prx IIE 50 ng, G6PDH1 130 ng) were ligated with Quick ligase. Half the volume of ligation mixture was used to transform 50 μL of *ECOS* Competent *E. coli* DH5α chemically competent cells.

^*a^The cloning efficiencies are represented as “the number of clones with the confirmed correct length of insert DNA by colony-PCR/number of colonies subjected to colony PCR”.

**Figure 3:**
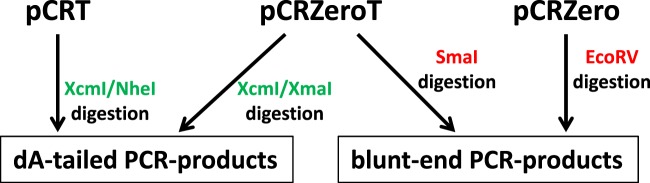
Selection of pCRT, pCRZero and pCRZeroT vectors for PCR cloning. pCRT and pCRZeroT can be used for TA cloning, whereas pCRZero and pCRZeroT can be used for blunt-end cloning. The pCRZeroT vector is compatible for both TA cloning and blunt-end cloning of PCR products. When blunt-end PCR products are directly cloned into non-digested vectors, SmaI (for pCRZeroT) or EcoRV (for pCRZero) can be selected, depending on the restriction sites present in the PCR product. Restriction enzymes to produce T-overhang for T-vectors are indicated by green letters. Restriction enzymes to produce blunt-end of vectors are indicated by red letters.

The three-types of vectors developed in this study worked well in both dA-tailed and blunt-end PCR cloning; the features of the three vectors are summarized in Fig. 3. The pCRT and pCRZeroT vectors are useful for TA cloning using XcmI digestion. Double digestion with XcmI/NheI (for pCRT) or XcmI/XmaI (for pCRZeroT) reduced the formation of background colonies due to empty vectors, and improved the rate of white colony formation. The pCRZero and pCRZeroT vectors are useful for blunt-end cloning using single digestion with EcoRV (for pCRZero) or SmaI (for pCRZeroT). This system does not require any additional reagents, such as IPTG/X-gal, and the procedure used with system is simple to follow.

Commercially available T-vectors and blunt-end vectors are generally expensive compared with in-house prepared vectors. For example, the cost of commercially available T-vectors from Promega, Novagen, and Thermo Fisher Scientific were in the range of $8.1 to $14.9 per reaction and that of commercially available blunt-end vectors with the *E. coli*-lethal *ccdB* gene from Thermo Fisher Scientific was ~$18 per reaction. In contrast, the preparation costs of T-vectors from pCRT and pCRZeroT and blunt-end vectors from pCRZero and pCRZeroT were ~$0.06 per reaction, including the cost of the medium required to proliferate the plasmid-carrying *E. coli* cells, the plasmid midi-kit for preparation of plasmids, restriction enzymes for linearization of vectors, and the DNA-purification kit for purification of linearized vectors.

### Direct PCR cloning into non-digested vectors using the Golden Gate reaction

**Figure 4:**
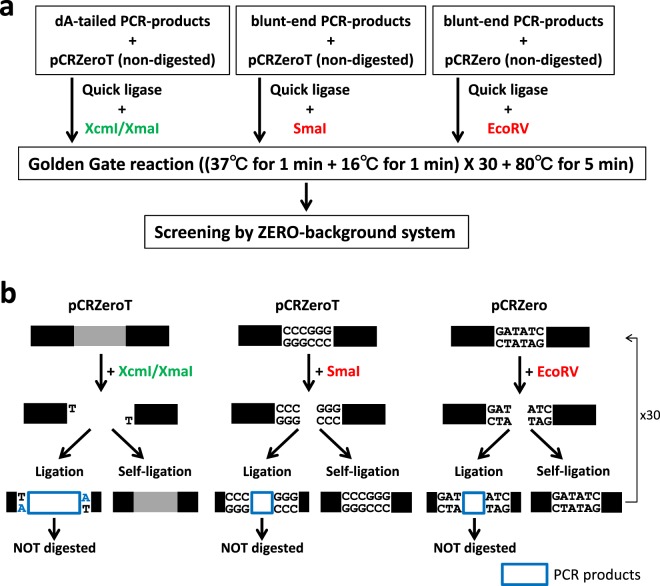
Overview of PCR cloning into non-digested circular vectors using the Golden Gate reaction. (**a**) Outline of PCR cloning into non-digested vectors. (**b**) Reaction cycle of Golden Gate reaction. Non-digested vector and PCR products were ligated in the presence of both ligase and indicated restriction enzymes and used in the following Golden Gate reaction cycle: (37 °C for 1 min + 16 °C for 1 min) × 30 cycles, then 80 °C for 5 min. Restriction enzymes to produce T-overhang for T-vectors are indicated by green letters. Restriction enzymes to produce blunt-end of vectors are indicated by red letters.

To develop a method that exploits the features of *ccdB-*dependent lethality, I attempted ligation with PCR products and non-digested vectors, applying the Golden Gate reaction that simultaneously digests and ligates vectors and PCR products (Fig. 4a,b)^22,23^. At the first step, pCRZero and pCRZeroT are digested by each restriction enzyme, and PCR products are inserted into each cloning site of the vectors. At the next step, vectors that contain ligated PCR-products are not digested, as the restriction site is destroyed during the ligation; in contrast, empty vectors remain susceptible to digestion (Fig. 4b). By repeating the Golden Gate reaction, the PCR cloning efficiencies increase, because *E. coli* DH5α carrying empty pCRZero and pCRZeroT vectors are unable to form colonies (Fig. 2 and Table 3). The dA-tailed PCR products of G6PDH1 (1.6 kbp) were cloned into T-overhangs of pCRZeroT with high efficiencies (>87%). The blunt-end PCR-products of G6PDH1 (1.6 kbp) and Prx IIE (0.6 kbp) were also cloned into a blunt-end site in pCRZero or pCRZeroT (Table 3) with efficiencies >95%. These high cloning efficiencies were comparable to those of a commercially available vector, pZErO2.1 (Thermo Fisher Scientific) containing the *E. coli-*lethal *ccdB* gene. Furthermore, colony formation rates were similar for both pre-digested vectors and non-digested vectors when using Golden Gate reaction (Fig. S1). However, this system requires that the PCR fragments do not contain the restriction enzyme site that is exploited for digestion of the vector. The end user can therefore choose between pCRZero and pCRZeroT based on a survey of restriction enzyme sites in their PCR products.

**Table 3:** Direct PCR cloning into non-digested vectors using the Golden Gate reaction.

vector	Restriction enzyme (Buffer)	Insert	White colonies (%)^*a^	Cloning efficiencies^*b^
pCRZeroT	XcmI/XmaI (CutSmart)	G6PDH1	100.0 ± 0.0	21/24
pCRZeroT	SmaI (CutSmart)	G6PDH1	—	24/24
pCRZero	EcoRV (CutSmart)	Prx IIE	—	23/24
pZErO2.1	EcoRV (CutSmart)	Prx IIE	—	24/24

The PCR products (G6PDH1; 1.6 kbp or Prx IIE; 0.6 kbp) were purified by a Gel/PCR Extraction Kit. The pCRZeroT, pCRZero, or pZErO2.1 (Non-digested, 50 ng) and the purified blunt-end PCR products (G6PDH1 130 ng or Prx IIE 50 ng) were ligated with Quick ligase and each restriction enzyme in the Golden Gate reaction. Half of the ligation mixture volume was used to transform 50 μL of *ECOS* Competent *E. coli* DH5α chemically competent cells. Plasmid pZErO2.1 was used as a control for commercially available vectors, and positive clones in pZErO2.1 were screened on an LB agar plate containing kanamycin (50 μg/mL).

^*a^The fraction of white colonies as a percentage of total colonies was calculated and expressed as mean percentage ± standard deviation of three independent experiments.

^*b^The cloning efficiencies are represented as “the number of clones with the confirmed correct length of insert DNA by colony PCR/number of colonies subjected to colony PCR”.

### Evaluation of a rapid protocol without purification steps in PCR cloning

**Figure 5:**
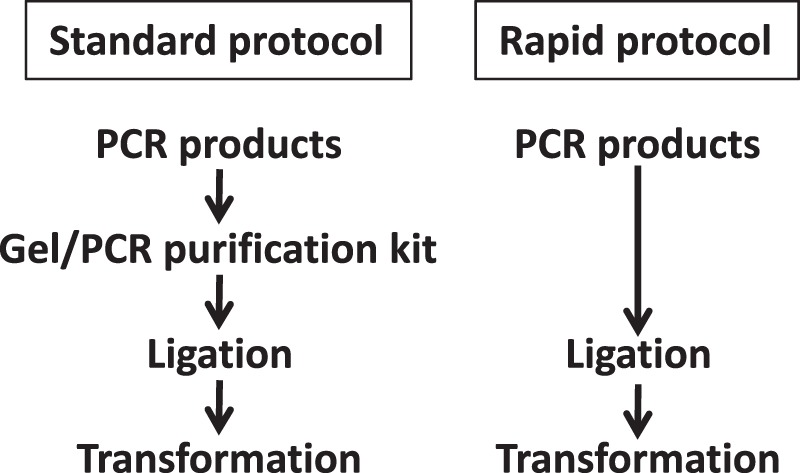
Outline of the PCR cloning processes of standard and rapid protocols.

Standard PCR cloning protocols generally use purified PCR products. Indeed, the three vectors developed in this study exhibited high cloning efficiencies using purified PCR fragments. To determine whether the procedure could be simplified even further, I determined whether colony-formation rate and cloning efficiencies were also acceptable when unpurified PCR products were used for both TA cloning and blunt-end cloning (Fig. 5). Strikingly, colony-formation rate and cloning efficiencies were almost identical for unpurified and purified PCR fragments when pCRZeroT was used for TA cloning and blunt-end cloning (Table 4). Therefore, purification of PCR products is not required in this ZERO-background cloning system. Moreover, the ratio of white colonies obtained using pCRZeroT as a T-vector was 100%, whereas that using pGEM-T Easy, a commercially available T-vector, was 10.8 ± 2.4% (−Purification) and 17.7 ± 1.8% (+Purification). When pCRZeroT was used as a T-vector, blue/white selection by IPTG/X-gal was not required.

**Table 4:** Evaluation of a simple protocol without purification of PCR products in PCR cloning.

Vector (Restriction enzymes for linearization)	Purification	White colonies (%)^*a^	Number of colonies^*b^	Cloning efficiencies^*c^
pCRZeroT (XcmI/XmaI)	−	100.0 ± 0.0	8.0 ± 5.6	15/24
pCRZeroT (XcmI/XmaI)	+	100.0 ± 0.0	31.7 ± 7.4	17/24
pCRZeroT (SmaI)	−	−	483.7 ± 43.6	22/24
pCRZeroT (SmaI)	+	−	673.7 ± 55.5	23/24
pGEM-T Easy	−	10.8 ± 2.4	30.0 ± 10.5	17/24
pGEM-T Easy	+	17.7 ± 1.8	112.7 ± 12.1	21/24

The G6PDH1 (1.6 kbp) gene was amplified by KAPATaq EXtra DNA polymerase (for pCRZeroT (XcmI/XmaI)) or Tks Gflex DNA polymerase (for pCRZeroT (SmaI)) in a 50-μL reaction. pCRZeroT was linearized by digestion with the indicated restriction enzymes, and purified by a Gel/PCR Extraction Kit. The linearized pCRZeroT (50 ng) and each of the PCR products (±purification, 1/20 volume) were then ligated. Two and a half microliters of the 50-μL PCR reaction (Purification (−)) or 1 μL of 20 μL purified PCR-products (Purification (+)) was used for ligation. Three tenths of the ligation reaction volume was used to transform 30 μL of *ECOS* Competent *E. coli* DH5α chemically competent cells. Commercially available T-vector pGEM-T Easy was used as a control.

^*a^The fraction of white colonies as a percentage of total colonies was calculated and expressed as mean percentage ± standard deviation of three independent experiments.

^*b^The number of colonies is the mean ± standard deviation of three independent experiments. When pCRZeroT (XcmI/XmaI) and pGEM-T Easy were used as T-vectors, the number of colonies was represented as the number of white colonies.

^*c^The cloning efficiencies are represented as “the number of clones with the confirmed correct length of insert DNA by colony PCR/number of white colonies subjected to colony PCR”.

### Blunting and blunt-end cloning of the dA-tailed DNA fragments in blunt-end positive-selection system using the *ccdB* gene

**Figure 6:**
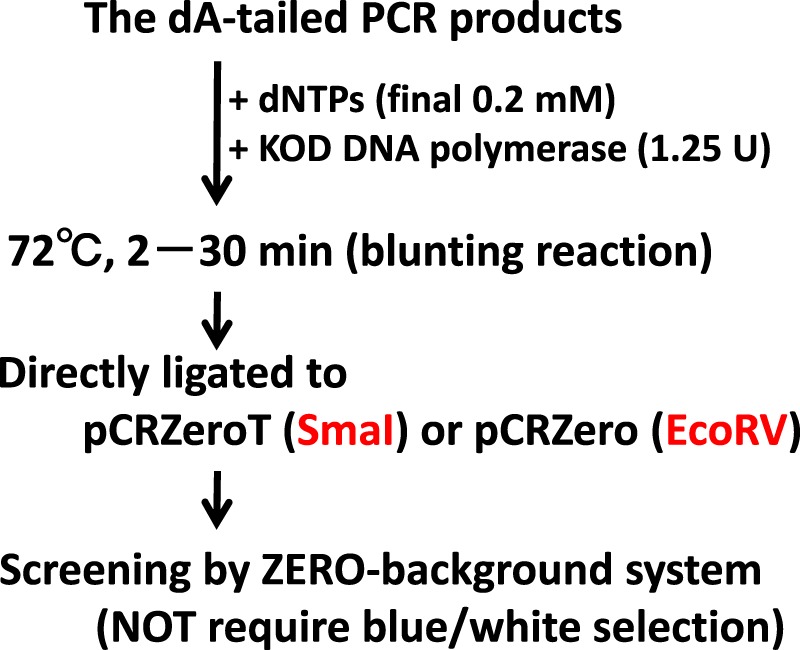
Description of the blunting reaction and blunt-end cloning process for dA-tailed PCR-products. Restriction enzymes to produce blunt-ends within vectors are indicated by red letters.

Although TA cloning is a standard cloning method for dA-tailed PCR-products, it requires blue/white selection using IPTG and X-gal in order to maximize cloning efficiency. The selection procedure is long, since time is required for expression of lacZα protein; furthermore, the system is prone to false positives as a result of insufficient lacZα expression. Hi-fidelity thermostable DNA polymerases such as KOD DNA polymerase, have 3′ to 5′ exonuclease activity for proofreading, and can delete the dA added at the 3′ terminus of the dA-tailed PCR-fragments^4^. To estimate blunting activities of the dA-tailed PCR-products polished by KOD DNA polymerase, PCR fragments amplified by KAPATaq EXtra DNA polymerase were directly treated with KOD DNA polymerase in order to blunt the dA-tailed PCR-fragments (Fig. 6). The 3′-exonuclease activity of DNA polymerase polishes the ends of the PCR fragments in the presence of dNTPs^25^. When the dA-tailed PCR-products were ligated to pCRZeroT (SmaI), no positive clones were obtained (Table 5). By contrast, colony formation rates increased in a blunting reaction-dependent manner, and cloning efficiencies were 58.3% and 66.7% after 2 and 30 min treatment with high-fidelity KOD DNA polymerase, respectively.

**Table 5:** Blunting and blunt-end cloning of the dA-tailed DNA fragments in the blunt-end positive-selection system using the *ccdB* gene.

Vector (Restriction enzyme for linearization)	Blunting reaction	Number of colonies^*a^	Cloning efficiencies^*b^
pCRZeroT (SmaI)	None	11.3 ± 3.1	0/24
pCRZeroT (SmaI)	KOD DNA pol. 2 min	59.7 ± 22.0	14/24
pCRZeroT (SmaI)	KOD DNA pol. 30 min	84.7 ± 7.1	16/24

The pCRZeroT was linearized by blunt-end digestion with SmaI, and purified by a Gel/PCR Extraction Kit. The G6PDH1 (1.6 kbp) gene was amplified by KAPATaq EXtra DNA polymerase in a 50 μL reaction volume. The dNTPs (final 0.2 mM) and KOD DNA polymerase (1.25 U) were directly added to 50 μL of unpurified dA-tailed PCR-products. Blunting reactions were performed for 2 or 30 min at 72 °C. pCRZeroT (SmaI) (50 ng) and 2.5 μL of the 50 μL PCR reaction were then ligated. Three tenths of the ligation mixture were used to transform 30 μL of *ECOS* Competent *E. coli* DH5α chemically competent cells.

^*a^The number of colonies is the mean ± standard deviation of three independent experiments.

^*b^The cloning efficiencies are represented as “the number of clones with the confirmed correct length of insert DNA by colony PCR/number of colonies subjected to colony PCR”.

## Conclusion

Three types of PCR cloning vectors were developed in this study. The pCRT and pCRZeroT vectors can be used for TA cloning, and are far less susceptible to false positives due to the empty vector digestion strategy using additional restriction enzymes I developed. The pCRZero and pCRZeroT can be used for blunt-end cloning by exploiting *ccdB-*dependent lethality. As an application of the ZERO-background system, I developed a direct PCR cloning method with the PCR products and non-digested vectors using a Golden Gate reaction. This system does not require pre-digestion of cloning vectors, and can therefore be used when the amount of vector is limiting, since purification is not required. In addition, researchers can select either pCRZero and pCRZeroT based on the restriction sites that are present in their PCR products. Furthermore, the PCR cloning procedure is simplified by elimination of purification steps and blunting of the dA-tailed PCR products. Blue/white selection with IPTG/X-gal can be omitted by using the blunt-end cloning strategy of the ZERO-background system. The plasmids, along with maps and sequences, can be accessed via Addgene (https://www.addgene.org/) as IDs 120274 (pCRT), 120275 (pCRZero) and 120276 (for pCRZeroT).

## Methods

### Materials

Plasmid pUC18 (Takara-Bio, Otsu, Japan) was used to construct pCRT, pCRZero and pCRZeroT vectors. Plasmid pZErO2.1 (Thermo Fisher Scientific, Carlsbad, CA) served as the *ccdB* gene donor vector. Plasmid pZErO2.1 was also used as a control vector for blunt-end cloning using the lethal *ccdB* gene. T-vector pGEM-T Easy (Promega, Madison, WI) was used as a control for commercially available T-vectors. *E. coli* JM109^26^, XL10-Gold (Agilent Technologies, Santa Clara, CA) or NEB Turbo (NEB, Ipswich, MA) that contain the F-plasmid, were used to prepare pCRZero and pCRZeroT plasmids expressing the *ccdB* gene^14,27^. *E. coli* DH5α (a *ccdB*-sensitive strain)^28^ was used for positive selection during PCR cloning with either pCRZero and pCRZeroT, which express the *ccdB* gene in the absence of ligated PCR products. The *Arabidopsis* type II peroxiredoxin E (Prx IIE, 0.6 kbp, AT3G52960)^29,30^ and chloroplast glucose-6-phosphate dehydrogenase 1 (G6PDH1, 1.6 kbp, AT5G35790)^31^ genes were used as sources for PCR amplification.

### In silico cloning

Serial Cloner 2.6 (http://serialbasics.free.fr/Serial_Cloner.html) was used to analyze sequence data, design primers, and design cloning strategies. ApE v2.0.55 (http://jorgensen.biology.utah.edu/wayned/ape/) was used to generate the plasmid maps shown in the figures. Additionally, PlasMapper (http://wishart.biology.ualberta.ca/PlasMapper/)^32^ was used to generate the plasmid text maps in Supplementary Dataset S1.

### Construction of pCRT, pCRZero, and pCRZeroT vectors

In pCRT, two complementary single strand DNA oligonucleotides containing an XcmI cassette (42-mer) were inserted in order to generate the T-overhang of the T-vector (Table S1) for seamless ligation cloning extract (SLiCE)-cloning^33,34^. Two single strand oligonucleotides, pUC18TXcmIcassette42merSLiCE-F (10 μM) and pUC18TXcmIcassette42merSLiCE-R (10 μM) in 100 μL solution containing 10 mM Tris-HCl (pH 8.0) and 0.1 mM EDTA in a 1.5 mL microtube, were denatured at 85 °C in a 500-mL glass beaker filled with water, and the sample was left for 150 min until it reached room temperature. Annealed XcmI-cassette oligonucleotides for SLiCE-cloning (0.3 μL) were inserted into a XmaI site (C|CCGGG) in pUC18 (25 ng), using the SLiCE from a laboratory *E. coli* strain, JM109^35^. In pCRZero, the *ccdB* gene was amplified by KOD DNA polymerase (Toyobo, Osaka, Japan)^4^ with pUC18Zero-F and pUC18Zero-R primers (Table S1), using pZErO2.1 plasmid as the template. Amplified DNA fragments were inserted into EcoRI and HindIII sites in pUC18, by seamless ligation of the SLiCE from a laboratory *E. coli* strain, JM109^34,36^. In pCRZeroT, *ccdB* was amplified as two fragments by KOD DNA polymerase using two pairs of primers (pUCR18ZeroT-Xcm-CcdB-NF/pUC18ZeroT-CcdB-NRsma and pUC18ZeroT-CcdB-CFsma/pUC18ZeroT-Xcm-CcdB-CR2); this introduced a SmaI site (CCC|GGG) for blunt-end cloning into the *ccdB* gene (Table S1). The mutation that substituted CCGGGG to CCCGGG allowed the introduction of a SmaI site without changing the amino acid sequence of CcdB. Two amplified DNA fragments were simultaneously inserted into EcoRI and HindIII sites in pUC18 using the SLiP-method^33,35^. These plasmids were used to transform the *ccdB*-resistant strains XL10-Gold and NEB Turbo prior to screening by colony PCR (Table S1)^37–39^. The DNA sequence of all plasmids was confirmed by sequencing^40^.

### Preparation of linearized pCRT, pCRZero, and pCRZeroT vectors for PCR cloning

The pCRZero and pCRZeroT plasmids were amplified in *ccdB*-gene resistant strains carrying the F-plasmid, such as JM109, XL10-Gold and NEB Turbo^14,27^. pCRT was digested by XcmI for TA cloning. As an additional restriction enzyme, NheI was also added to reduce background colony formation due to empty vector. pCRZero was digested by EcoRV in order to generate blunt ends for cloning. pCRZeroT was digested by XcmI for use in TA cloning. As an additional restriction enzyme, XmaI was also added to reduce background colony formation. pCRZeroT for use in blunt-end cloning was digested by SmaI. All linearized vectors were purified by a FastGene Gel/PCR Extraction Kit (Nippon Genetics, Tokyo, Japan); this procedure did not require separation by agarose gel electrophoresis.

### Amplification of dA-tailed or blunt-end PCR-products by thermostable DNA polymerase

Prx IIE (0.6 kbp, AT3G52960) and G6PDH1 (1.6 kbp, AT5G35790) genes were amplified with specific primers (Table S2)^33^. The dA-tailed PCR-products were amplified by KAPATaq EXtra DNA polymerase (KAPA Biosystems, Wilmington, MA), and the blunt-end PCR-products were amplified by Tks Gflex DNA polymerase (Takara-Bio, Otsu, Japan). When purification was required for PCR cloning, both PCR products were purified using a FastGene Gel/PCR Extraction Kit without prior separation via agarose gel-electrophoresis.

### Evaluation of cloning efficiency by pCRT and pCRZeroT for TA cloning of PCR products

Cloning efficiencies with the pCRT and pCRZeroT vectors (in which a T-overhang was produced by XcmI digestion) were evaluated using dA-tailed PCR-products. Linearized pCRT or pCRZeroT (50 ng), and purified dA-tailed PCR products (G6PDH1, 130 ng) were mixed with a vector:insert molar ratio of 1:5, and then ligated for 30 min at 16 °C using ligation convenient kit (Nippon Gene, Tokyo, Japan) or Quick Ligation Kit (NEB; used throughout unless stated otherwise). After the reaction the ligation mixtures were used directly to transform *ECOS* Competent *E. coli* DH5α chemically competent cells (100 μL, ~1 × 10^7^ CFUs/μg of pUC19 DNA) as described in the Figure and Table Legends. The percentage of white colonies was determined by blue/white selection using α-complementation mediated by the *lac*Z gene. The cloning efficiency of PCR products was estimated by colony PCR, using the primers described in Table S3. For this and all subsequent sections, the number of colonies was represented as colony number on an LB agar plate containing ampicillin (final 100 μg/mL). Similarly, cloning efficiencies for the insert DNA were given as the ratio of colonies with an insert of the confirmed correct length as estimated by colony PCR.

### Evaluation of cloning efficiency by pCRZero and pCRZeroT for blunt-end cloning of PCR products

Cloning efficiency of pCRZero and pCRZeroT vectors linearized to blunt-ends with EcoRV (for pCRZero) or SmaI (for pCRZeroT), were evaluated using blunt-end PCR-products amplified by Tks Gflex DNA polymerase (Takara-Bio, Otsu, Japan). Linearized pCRZero or pCRZeroT (50 ng), and blunt-end PCR products (50 ng (Prx IIE) or 130 ng (G6PDH1)) were mixed with a vector:insert molar ratio of 1:5, and then ligated for 30 min at 16 °C. After the reaction, ligation mixtures were used to transform *ECOS* Competent *E. coli* DH5α cells as described in the Figure and Table Legends. The cloning efficiency of PCR products was estimated by colony PCR, using primers described in Table S3.

### Direct PCR cloning into non-digested vectors carrying the *ccdB* gene using the Golden Gate reaction

Prx IIE (0.6 kbp) and G6PDH1 (1.6 kbp) genes were templates for PCR cloning (Table S2). The dA-tailed DNA fragments were amplified by KAPATaq EXtra DNA polymerase for pCRZeroT (XcmI/XmaI), and the blunt-end DNA fragments were amplified by Tks Gflex DNA polymerase for pCRZero (EcoRV) and pCRZeroT (SmaI). All PCR-amplified DNA fragments were purified by a FastGene Gel/PCR Extraction Kit without separation of agarose gel-electrophoresis. Non-digested vectors (50 ng) and, PCR products (50 ng (Prx IIE) or 130 ng (G6PDH1)) were ligated. Ligation for non-digested vectors using the Golden Gate reaction was performed as following: 1 min at 37 °C followed by 1 min at 16 °C, both repeated 30 times followed by 5 min at 80 °C, in a total volume of 10 μL containing CutSmart Buffer (2×), 1 mM ATP and restriction enzymes (XcmI 10 U, XmaI 5 U, SmaI 10 U or EcoRV 15 U). As a control experiment, pre-digested vectors (50 ng) and PCR products (50 ng (Prx IIE) or 130 ng (G6PDH1)) were ligated. Ligation of pre-digested vectors and the PCR products was performed for 30 min at 16 °C in a total volume of 10 μL containing CutSmart Buffer (2×), 1 mM ATP. Then, one and a half microliters of the ligation mix was used to transform 20 μL of *ECOS* Competent *E. coli* DH5α cells. The cloning efficiency of PCR products was estimated by colony PCR using the primers listed in Table S3.

### PCR cloning into the pCRZeroT vector of unpurified or purified insert DNA fragments

G6PDH1 (1.6 kbp) gene was amplified in a 50-μL PCR mixture by KAPATaq EXtra DNA polymerase (for pCRZeroT (XcmI/XmaI)) or Tks Gflex DNA polymerase (for pCRZeroT (SmaI)). Two and a half microliters (1/20 volume) of the 50-μL PCR mixture and linearized vector (50 ng) were directly ligated in a 10-μL ligation solution, without purification of PCR products. When purification was required, PCR mixtures (50 μL) were processed with a FastGene Gel/PCR Extraction Kit, and eluted in 20 μL elution buffer. One microliter (1/20 volume) of purified PCR products and linearized vector (50 ng) were ligated in 10-μL ligation solutions. After the reaction, ligation mixtures were used to transform *ECOS* Competent *E. coli* DH5α cells as described in the Figure and Table Legends.

### Blunting reaction of the dA-tailed PCR fragments and blunt-end cloning at a SmaI site in pCRZeroT

dNTPs (final 0.2 mM) and KOD DNA polymerase (1.25 U; this enzyme has 3′ to 5′ exonuclease activity) were directly added to the reaction mixture (50 μL) after PCR reaction, for blunting of the dA-tailed PCR fragments (G6PDH1; 1.6 kbp) amplified by KAPATaq EXtra DNA polymerase. The mixtures were incubated at 72 °C for 2 min or 30 min, and used directly to transform *ECOS* Competent *E. coli* DH5α cells without any purification step. Two and a half microliters (1/20 volume) of the 50-μL PCR mixture and linearized vector (50 ng) were then ligated for 30 min at 16 °C in a total volume of 10 μL containing CutSmart Buffer (2×) and 1 mM ATP. Three microliters of the 10 μL ligation mixtures was used to transform 30 μL of *ECOS* Competent *E. coli* DH5α cells.

## Supplementary information

Supplementary information

Dataset S1
