# Supplementary material for: A novel series of high-efficiency vectors for TA cloning and blunt-end cloning of PCR products
Source: Sci Rep. 2019 Apr 23;9:6417. doi: 10.1038/s41598-019-42868-6 (PMC6478821; doi:10.1038/s41598-019-42868-6)
Supplement: Supplementary file 1 — Supplementary information [file 41598_2019_42868_MOESM1_ESM.pdf]

A novel series of high-efficiency vectors for TA cloning and blunt-end cloning of PCR products

Ken Motohashi[a,b]*
[a]Department of Frontier Life Sciences, Faculty of Life Sciences, Kyoto Sangyo University, Kamigamo Motoyama, Kita-ku, Kyoto 603-8555, Japan
[b]Center for Ecological Evolutionary Developmental Biology, Kyoto Sangyo University, Kamigamo Motoyama, Kita-Ku, Kyoto 603-8555, Japan

*Corresponding author: Ken Motohashi
E-mail: motohas@cc.kyoto-su.ac.jp

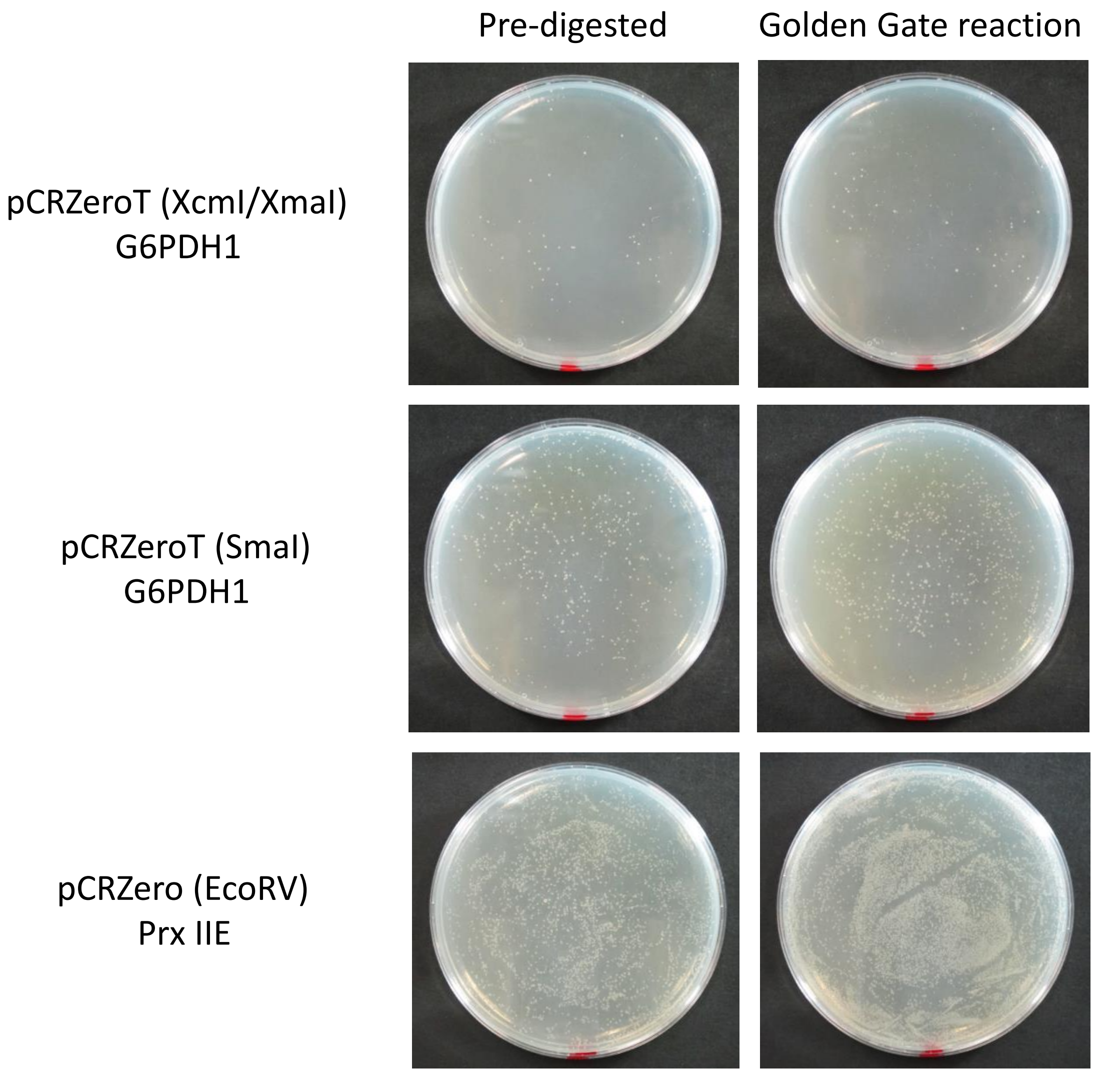

Supplementary Figure S1 Colony formation following PCR cloning into pre-digested vectors using a standard method and into non-digested vectors using the Golden Gate reaction.

The G6PDH1 and Prx IIE genes were amplified by KAPATaq EXtra DNA polymerase for pCRZeroT (XcmI/XmaI), or by Tks Gflex DNA polymerase for pCRZero (EcoRV) and pCRZeroT (SmaI). Purified pre-digested or non-digested vectors (50 ng) and purified PCR products (130 ng (G6PDH1) or 50 ng (Prx IIE)), were then ligated. Ligation for pre-digested vectors and the PCR products was performed for 30 min at 16° C in a total volume of 10 μL containing CutSmart Buffer (2×) and 1 mM ATP. Ligation for non-digested vectors using Golden Gate reaction was performed as following: 1 min at 37° C followed by 1 min at 16° C, both repeated 30 times, and then 5 min at 80° C, in total 10 μL containing CutSmart Buffer (2×), 1 mM ATP and restriction enzymes (XcmI 10U, XmaI 5U, SmaI 10U, or EcoRV 15U). One and a half microliters of the 10 μL ligation mix was used to transform 20 μL of *ECOS* Competent *E. coli* DH5α chemically competent cells.

Table S1 Oligonucleotides for vector constructions

Primer	Sequence (5′ to 3′)
**For pCRT**	
pUC18TXcmIcassette42merSLiCE-F	ttcgagctcggtacccaatacttgtatggagacgctagcgtctccatacaagtattggggatcctctagagt
pUC18TXcmIcassette42merSLiCE-R	actctagaggatccccaatacttgtatggagacgctagcgtctccatacaagtattgggtaccgagctcgaa
**For pCRZero**	
pUC18Zero-F	cagctatgaccatgattacgc
pUC18Zero-R	aacgacggccagtgccaagttatattccccagaacatcag
**For pCRZeroT**	
pUC18ZeroT-Xcm-CcdB-NF	gctatgaccatgattacgaattcgccaatacttgtatggcagtttaaggtttacacctat
pUC18ZeroT-CcdB-NRsma	accatccgtcgcccgggcgtgtcaataatatcact
pUC18ZeroT-CcdB-CFsma	cgggcgacggatggtgatccc
pUC18ZeroT-Xcm-CcdB-CR2	aacgacggccagtgccaagcttccaatacttgtatggttatattccccagaacatca
**For colony-PCR & DNA sequence**	
pUC18-118F	tacactttatgcttccggctcgta
pUC18-118R	gaaagggggatgtgctgcaaggcg
pUC18-118R2	atcaggcgccattcgccattcagg

XcmI cassette (42-mer) is indicated by red letters. Introduced restriction enzyme cleavage sites are underlined (XcmI (CCAXXXXX|XXXXTGG) and SmaI (CCC|GGG)).

Table S2 Oligonucleotides for PCR-amplification of Prx IIE and G6PDH1 genes

Primer	Sequence (5′ to 3′)
**For Prx IIE (AT3G52960)**	
PrxIIE_10F	aggagatatacatATGgcctccatttccgtcgga
PrxIIE_10R	ggtggtggtgctcgagTCAgagagctttaagcatatc
**For G6PDH1 (AT5G35790)**	
G6PDH_10F	aggagatataccATGgccgagaaacattctcag
G6PDH_10R	ggtggtggtgctcgagTCAagcttctccaagatctcccca

Start and Stop codons are indicated by capital letters. Introduced restriction enzyme cleavage sites are underlined (NdeI, NcoI and XhoI).

Table S3 Oligonucleotides for colony PCR in PCR cloning

Primer[*1]	Sequence (5′ to 3′)[*2]
**For pCRT**	
M13F	gagcggataacaatttcacacagg
M13R	cgccagggttttcccagtcacgac
**For pCRZero**	
SP6	agctatttaggtgacactatagaa
pZErO_Rs2	ggccagtgaattgtaatacgactt
**For pCRZeroT in TA-cloning**	
M13F	gagcggataacaatttcacacagg
M13R	cgccagggttttcccagtcacgac
**For pCRZeroT in blunt-end cloning**	
pCRZeroTccdF	cagtttaaggtttacacctataaa
pCRZeroTccdR	tcatccccgatatgcaccaccggg
**For pGEM-T Easy**	
pTAKN2_T7P	tgtaatacgactcactatagggg
SP6	agctatttaggtgacactatagaa
**For pZErO2.1**	
SP6	agctatttaggtgacactatagaa
pZErO_Rs2	ggccagtgaattgtaatacgactt

[*1] These primers are also adequate as the sequencing primers.

[*2] Oligonucleotides shown in Table S3 are also indicated in the text maps of three plasmids developed in this study (Supplementary Dataset S1).
