# Supplementary material for: A novel series of high-efficiency vectors for TA cloning and blunt-end cloning of PCR products
Source: Sci Rep. 2019 Apr 23;9:6417. doi: 10.1038/s41598-019-42868-6 (PMC6478821; doi:10.1038/s41598-019-42868-6)
Supplement: Supplementary file 2 — Dataset S1 [file 41598_2019_42868_MOESM2_ESM.zip › Supplementary_DatasetS1/pCRT_txt_map.docx]

>pCRT_2728bp

1 GACGAAAGGGCCTCGTGATACGCCTATTTTTATAGGTTAATGTCATGATAATAATGGTTT 60

CTGCTTTCCCGGAGCACTATGCGGATAAAAATATCCAATTACAGTACTATTATTACCAAA

61 CTTAGACGTCAGGTGGCACTTTTCGGGGAAATGTGCGCGGAACCCCTATTTGTTTATTTT 120

GAATCTGCAGTCCACCGTGAAAAGCCCCTTTACACGCGCCTTGGGGATAAACAAATAAAA

amp prom(131,159)>>>

|

121 TCTAAATACATTCAAATATGTATCCGCTCATGAGACAATAACCCTGATAAATGCTTCAAT 180

AGATTTATGTAAGTTTATACATAGGCGAGTACTCTGTTATTGGGACTATTTACGAAGTTA

amp marker(201,1061)>>>

|

181 AATATTGAAAAAGGAAGAGTATGAGTATTCAACATTTCCGTGTCGCCCTTATTCCCTTTT 240

TTATAACTTTTTCCTTCTCATACTCATAAGTTGTAAAGGCACAGCGGGAATAAGGGAAAA

241 TTGCGGCATTTTGCCTTCCTGTTTTTGCTCACCCAGAAACGCTGGTGAAAGTAAAAGATG 300

AACGCCGTAAAACGGAAGGACAAAAACGAGTGGGTCTTTGCGACCACTTTCATTTTCTAC

301 CTGAAGATCAGTTGGGTGCACGAGTGGGTTACATCGAACTGGATCTCAACAGCGGTAAGA 360

GACTTCTAGTCAACCCACGTGCTCACCCAATGTAGCTTGACCTAGAGTTGTCGCCATTCT

361 TCCTTGAGAGTTTTCGCCCCGAAGAACGTTTTCCAATGATGAGCACTTTTAAAGTTCTGC 420

AGGAACTCTCAAAAGCGGGGCTTCTTGCAAAAGGTTACTACTCGTGAAAATTTCAAGACG

421 TATGTGGCGCGGTATTATCCCGTATTGACGCCGGGCAAGAGCAACTCGGTCGCCGCATAC 480

ATACACCGCGCCATAATAGGGCATAACTGCGGCCCGTTCTCGTTGAGCCAGCGGCGTATG

481 ACTATTCTCAGAATGACTTGGTTGAGTACTCACCAGTCACAGAAAAGCATCTTACGGATG 540

TGATAAGAGTCTTACTGAACCAACTCATGAGTGGTCAGTGTCTTTTCGTAGAATGCCTAC

541 GCATGACAGTAAGAGAATTATGCAGTGCTGCCATAACCATGAGTGATAACACTGCGGCCA 600

CGTACTGTCATTCTCTTAATACGTCACGACGGTATTGGTACTCACTATTGTGACGCCGGT

601 ACTTACTTCTGACAACGATCGGAGGACCGAAGGAGCTAACCGCTTTTTTGCACAACATGG 660

TGAATGAAGACTGTTGCTAGCCTCCTGGCTTCCTCGATTGGCGAAAAAACGTGTTGTACC

661 GGGATCATGTAACTCGCCTTGATCGTTGGGAACCGGAGCTGAATGAAGCCATACCAAACG 720

CCCTAGTACATTGAGCGGAACTAGCAACCCTTGGCCTCGACTTACTTCGGTATGGTTTGC

721 ACGAGCGTGACACCACGATGCCTGTAGCAATGGCAACAACGTTGCGCAAACTATTAACTG 780

TGCTCGCACTGTGGTGCTACGGACATCGTTACCGTTGTTGCAACGCGTTTGATAATTGAC

781 GCGAACTACTTACTCTAGCTTCCCGGCAACAATTAATAGACTGGATGGAGGCGGATAAAG 840

CGCTTGATGAATGAGATCGAAGGGCCGTTGTTAATTATCTGACCTACCTCCGCCTATTTC

841 TTGCAGGACCACTTCTGCGCTCGGCCCTTCCGGCTGGCTGGTTTATTGCTGATAAATCTG 900

AACGTCCTGGTGAAGACGCGAGCCGGGAAGGCCGACCGACCAAATAACGACTATTTAGAC

901 GAGCCGGTGAGCGTGGGTCTCGCGGTATCATTGCAGCACTGGGGCCAGATGGTAAGCCCT 960

CTCGGCCACTCGCACCCAGAGCGCCATAGTAACGTCGTGACCCCGGTCTACCATTCGGGA

961 CCCGTATCGTAGTTATCTACACGACGGGGAGTCAGGCAACTATGGATGAACGAAATAGAC 1020

GGGCATAGCATCAATAGATGTGCTGCCCCTCAGTCCGTTGATACCTACTTGCTTTATCTG

1021 AGATCGCTGAGATAGGTGCCTCACTGATTAAGCATTGGTAACTGTCAGACCAAGTTTACT 1080

TCTAGCGACTCTATCCACGGAGTGACTAATTCGTAACCATTGACAGTCTGGTTCAAATGA

1081 CATATATACTTTAGATTGATTTAAAACTTCATTTTTAATTTAAAAGGATCTAGGTGAAGA 1140

GTATATATGAAATCTAACTAAATTTTGAAGTAAAAATTAAATTTTCCTAGATCCACTTCT

1141 TCCTTTTTGATAATCTCATGACCAAAATCCCTTAACGTGAGTTTTCGTTCCACTGAGCGT 1200

AGGAAAAACTATTAGAGTACTGGTTTTAGGGAATTGCACTCAAAAGCAAGGTGACTCGCA

pMB1 origin(1216,1835)>>>

|

1201 CAGACCCCGTAGAAAAGATCAAAGGATCTTCTTGAGATCCTTTTTTTCTGCGCGTAATCT 1260

GTCTGGGGCATCTTTTCTAGTTTCCTAGAAGAACTCTAGGAAAAAAAGACGCGCATTAGA

1261 GCTGCTTGCAAACAAAAAAACCACCGCTACCAGCGGTGGTTTGTTTGCCGGATCAAGAGC 1320

CGACGAACGTTTGTTTTTTTGGTGGCGATGGTCGCCACCAAACAAACGGCCTAGTTCTCG

1321 TACCAACTCTTTTTCCGAAGGTAACTGGCTTCAGCAGAGCGCAGATACCAAATACTGTTC 1380

ATGGTTGAGAAAAAGGCTTCCATTGACCGAAGTCGTCTCGCGTCTATGGTTTATGACAAG

1381 TTCTAGTGTAGCCGTAGTTAGGCCACCACTTCAAGAACTCTGTAGCACCGCCTACATACC 1440

AAGATCACATCGGCATCAATCCGGTGGTGAAGTTCTTGAGACATCGTGGCGGATGTATGG

AlwNI

|

1441 TCGCTCTGCTAATCCTGTTACCAGTGGCTGCTGCCAGTGGCGATAAGTCGTGTCTTACCG 1500

AGCGAGACGATTAGGACAATGGTCACCGACGACGGTCACCGCTATTCAGCACAGAATGGC

1501 GGTTGGACTCAAGACGATAGTTACCGGATAAGGCGCAGCGGTCGGGCTGAACGGGGGGTT 1560

CCAACCTGAGTTCTGCTATCAATGGCCTATTCCGCGTCGCCAGCCCGACTTGCCCCCCAA

1561 CGTGCACACAGCCCAGCTTGGAGCGAACGACCTACACCGAACTGAGATACCTACAGCGTG 1620

GCACGTGTGTCGGGTCGAACCTCGCTTGCTGGATGTGGCTTGACTCTATGGATGTCGCAC

1621 AGCTATGAGAAAGCGCCACGCTTCCCGAAGGGAGAAAGGCGGACAGGTATCCGGTAAGCG 1680

TCGATACTCTTTCGCGGTGCGAAGGGCTTCCCTCTTTCCGCCTGTCCATAGGCCATTCGC

1681 GCAGGGTCGGAACAGGAGAGCGCACGAGGGAGCTTCCAGGGGGAAACGCCTGGTATCTTT 1740

CGTCCCAGCCTTGTCCTCTCGCGTGCTCCCTCGAAGGTCCCCCTTTGCGGACCATAGAAA

1741 ATAGTCCTGTCGGGTTTCGCCACCTCTGACTTGAGCGTCGATTTTTGTGATGCTCGTCAG 1800

TATCAGGACAGCCCAAAGCGGTGGAGACTGAACTCGCAGCTAAAAACACTACGAGCAGTC

1801 GGGGGCGGAGCCTATGGAAAAACGCCAGCAACGCGGCCTTTTTACGGTTCCTGGCCTTTT 1860

CCCCCGCCTCGGATACCTTTTTGCGGTCGTTGCGCCGGAAAAATGCCAAGGACCGGAAAA

1861 GCTGGCCTTTTGCTCACATGTTCTTTCCTGCGTTATCCCCTGATTCTGTGGATAACCGTA 1920

CGACCGGAAAACGAGTGTACAAGAAAGGACGCAATAGGGGACTAAGACACCTATTGGCAT

1921 TTACCGCCTTTGAGTGAGCTGATACCGCTCGCCGCAGCCGAACGACCGAGCGCAGCGAGT 1980

AATGGCGGAAACTCACTCGACTATGGCGAGCGGCGTCGGCTTGCTGGCTCGCGTCGCTCA

1981 CAGTGAGCGAGGAAGCGGAAGAGCGCCCAATACGCAAACCGCCTCTCCCCGCGCGTTGGC 2040

GTCACTCGCTCCTTCGCCTTCTCGCGGGTTATGCGTTTGGCGGAGAGGGGCGCGCAACCG

2041 CGATTCATTAATGCAGCTGGCACGACAGGTTTCCCGACTGGAAAGCGGGCAGTGAGCGCA 2100

GCTAAGTAATTACGTCGACCGTGCTGTCCAAAGGGCTGACCTTTCGCCCGTCACTCGCGT

pUC18-118F (2146,2169)>

|

lac prom(2144,2173)>>>

| |

2101 ACGCAATTAATGTGAGTTAGCTCACTCATTAGGCACCCCAGGCTTTACACTTTATGCTTC 2160

TGCGTTAATTACACTCAATCGAGTGAGTAATCCGTGGGGTCCGAAATGTGAAATACGAAG

M13F(2186,2209)> lacZ-alpha(2218,2583)>>>

| |

2161 CGGCTCGTATGTTGTGTGGAATTGTGAGCGGATAACAATTTCACACAGGAAACAGCTATG 2220

M

GCCGAGCATACAACACACCTTAACACTCGCCTATTGTTAAAGTGTGTCCTTTGTCGATAC

ApoI

|

EcoRI SacI KpnI XcmI(2) NheI

| | | | |

2221 ACCATGATTACGAATTCGAGCTCGGTACCCCAatacttgtaTGGagacgctagcgtctCC 2280

T M I T N S S S V P Q Y L Y G D A S V S

TGGTACTAATGCTTAAGCTCGAGCCATGGGGTTATGAACATACCTCTGCGATCGCAGAGG

HincII HindIII

| |

XcmI(2) BamHI XbaI AccI PstI SphI

| | | || | | |

2281 AtacaagtatTGGGGGATCCTCTAGAGTCGACCTGCAGGCATGCAAGCTTGGCACTGGCC 2340

I Q V L G D P L E S T C R H A S L A L A

TATGTTCATAACCCCCTAGGAGATCTCAGCTGGACGTCCGTACGTTCGAACCGTGACCGG

< M13R(2377,2354)

|

2341 GTCGTTTTACAACGTCGTGACTGGGAAAACCCTGGCGTTACCCAACTTAATCGCCTTGCA 2400

V V L Q R R D W E N P G V T Q L N R L A

CAGCAAAATGTTGCAGCACTGACCCTTTTGGGACCGCAATGGGTTGAATTAGCGGAACGT

< pUC18-118R (2415,2392)

|

2401 GCACATCCCCCTTTCGCCAGCTGGCGTAATAGCGAAGAGGCCCGCACCGATCGCCCTTCC 2460

A H P P F A S W R N S E E A R T D R P S

CGTGTAGGGGGAAAGCGGTCGACCGCATTATCGCTTCTCCGGGCGTGGCTAGCGGGAAGG

NarI < pUC18-118R2 (2498,2475)

| |

2461 CAACAGTTGCGCAGCCTGAATGGCGAATGGCGCCTGATGCGGTATTTTCTCCTTACGCAT 2520

Q Q L R S L N G E W R L M R Y F L L T H

GTTGTCAACGCGTCGGACTTACCGCTTACCGCGGACTACGCCATAAAAGAGGAATGCGTA

NdeI

|

2521 CTGTGCGGTATTTCACACCGCATATGGTGCACTCTCAGTACAATCTGCTCTGATGCCGCA 2580

L C G I S H R I W C T L S T I C S D A A

GACACGCCATAAAGTGTGGCGTATACCACGTGAGAGTCATGTTAGACGAGACTACGGCGT

2581 TAGTTAAGCCAGCCCCGACACCCGCCAACACCCGCTGACGCGCCCTGACGGGCTTGTCTG 2640

*

ATCAATTCGGTCGGGGCTGTGGGCGGTTGTGGGCGACTGCGCGGGACTGCCCGAACAGAC

2641 CTCCCGGCATCCGCTTACAGACAAGCTGTGACCGTCTCCGGGAGCTGCATGTGTCAGAGG 2700

GAGGGCCGTAGGCGAATGTCTGTTCGACACTGGCAGAGGCCCTCGACGTACACAGTCTCC

2701 TTTTCACCGTCATCACCGAAACGCGCGA 2728

AAAAGTGGCAGTAGTGGCTTTGCGCGCT
