# Supplementary material for: A novel series of high-efficiency vectors for TA cloning and blunt-end cloning of PCR products
Source: Sci Rep. 2019 Apr 23;9:6417. doi: 10.1038/s41598-019-42868-6 (PMC6478821; doi:10.1038/s41598-019-42868-6)
Supplement: Supplementary file 2 — Dataset S1 [file 41598_2019_42868_MOESM2_ESM.zip › Supplementary_DatasetS1/pCRZero_txt_map.docx]

>pCRZero_3272bp

1 GACGAAAGGGCCTCGTGATACGCCTATTTTTATAGGTTAATGTCATGATAATAATGGTTT 60

CTGCTTTCCCGGAGCACTATGCGGATAAAAATATCCAATTACAGTACTATTATTACCAAA

61 CTTAGACGTCAGGTGGCACTTTTCGGGGAAATGTGCGCGGAACCCCTATTTGTTTATTTT 120

GAATCTGCAGTCCACCGTGAAAAGCCCCTTTACACGCGCCTTGGGGATAAACAAATAAAA

amp prom(131,159)>>>

|

121 TCTAAATACATTCAAATATGTATCCGCTCATGAGACAATAACCCTGATAAATGCTTCAAT 180

AGATTTATGTAAGTTTATACATAGGCGAGTACTCTGTTATTGGGACTATTTACGAAGTTA

amp marker(201,1061)>>>

|

181 AATATTGAAAAAGGAAGAGTATGAGTATTCAACATTTCCGTGTCGCCCTTATTCCCTTTT 240

TTATAACTTTTTCCTTCTCATACTCATAAGTTGTAAAGGCACAGCGGGAATAAGGGAAAA

241 TTGCGGCATTTTGCCTTCCTGTTTTTGCTCACCCAGAAACGCTGGTGAAAGTAAAAGATG 300

AACGCCGTAAAACGGAAGGACAAAAACGAGTGGGTCTTTGCGACCACTTTCATTTTCTAC

301 CTGAAGATCAGTTGGGTGCACGAGTGGGTTACATCGAACTGGATCTCAACAGCGGTAAGA 360

GACTTCTAGTCAACCCACGTGCTCACCCAATGTAGCTTGACCTAGAGTTGTCGCCATTCT

361 TCCTTGAGAGTTTTCGCCCCGAAGAACGTTTTCCAATGATGAGCACTTTTAAAGTTCTGC 420

AGGAACTCTCAAAAGCGGGGCTTCTTGCAAAAGGTTACTACTCGTGAAAATTTCAAGACG

421 TATGTGGCGCGGTATTATCCCGTATTGACGCCGGGCAAGAGCAACTCGGTCGCCGCATAC 480

ATACACCGCGCCATAATAGGGCATAACTGCGGCCCGTTCTCGTTGAGCCAGCGGCGTATG

481 ACTATTCTCAGAATGACTTGGTTGAGTACTCACCAGTCACAGAAAAGCATCTTACGGATG 540

TGATAAGAGTCTTACTGAACCAACTCATGAGTGGTCAGTGTCTTTTCGTAGAATGCCTAC

541 GCATGACAGTAAGAGAATTATGCAGTGCTGCCATAACCATGAGTGATAACACTGCGGCCA 600

CGTACTGTCATTCTCTTAATACGTCACGACGGTATTGGTACTCACTATTGTGACGCCGGT

601 ACTTACTTCTGACAACGATCGGAGGACCGAAGGAGCTAACCGCTTTTTTGCACAACATGG 660

TGAATGAAGACTGTTGCTAGCCTCCTGGCTTCCTCGATTGGCGAAAAAACGTGTTGTACC

661 GGGATCATGTAACTCGCCTTGATCGTTGGGAACCGGAGCTGAATGAAGCCATACCAAACG 720

CCCTAGTACATTGAGCGGAACTAGCAACCCTTGGCCTCGACTTACTTCGGTATGGTTTGC

721 ACGAGCGTGACACCACGATGCCTGTAGCAATGGCAACAACGTTGCGCAAACTATTAACTG 780

TGCTCGCACTGTGGTGCTACGGACATCGTTACCGTTGTTGCAACGCGTTTGATAATTGAC

781 GCGAACTACTTACTCTAGCTTCCCGGCAACAATTAATAGACTGGATGGAGGCGGATAAAG 840

CGCTTGATGAATGAGATCGAAGGGCCGTTGTTAATTATCTGACCTACCTCCGCCTATTTC

841 TTGCAGGACCACTTCTGCGCTCGGCCCTTCCGGCTGGCTGGTTTATTGCTGATAAATCTG 900

AACGTCCTGGTGAAGACGCGAGCCGGGAAGGCCGACCGACCAAATAACGACTATTTAGAC

901 GAGCCGGTGAGCGTGGGTCTCGCGGTATCATTGCAGCACTGGGGCCAGATGGTAAGCCCT 960

CTCGGCCACTCGCACCCAGAGCGCCATAGTAACGTCGTGACCCCGGTCTACCATTCGGGA

961 CCCGTATCGTAGTTATCTACACGACGGGGAGTCAGGCAACTATGGATGAACGAAATAGAC 1020

GGGCATAGCATCAATAGATGTGCTGCCCCTCAGTCCGTTGATACCTACTTGCTTTATCTG

1021 AGATCGCTGAGATAGGTGCCTCACTGATTAAGCATTGGTAACTGTCAGACCAAGTTTACT 1080

TCTAGCGACTCTATCCACGGAGTGACTAATTCGTAACCATTGACAGTCTGGTTCAAATGA

1081 CATATATACTTTAGATTGATTTAAAACTTCATTTTTAATTTAAAAGGATCTAGGTGAAGA 1140

GTATATATGAAATCTAACTAAATTTTGAAGTAAAAATTAAATTTTCCTAGATCCACTTCT

1141 TCCTTTTTGATAATCTCATGACCAAAATCCCTTAACGTGAGTTTTCGTTCCACTGAGCGT 1200

AGGAAAAACTATTAGAGTACTGGTTTTAGGGAATTGCACTCAAAAGCAAGGTGACTCGCA

pMB1 origin(1216,1835)>>>

|

1201 CAGACCCCGTAGAAAAGATCAAAGGATCTTCTTGAGATCCTTTTTTTCTGCGCGTAATCT 1260

GTCTGGGGCATCTTTTCTAGTTTCCTAGAAGAACTCTAGGAAAAAAAGACGCGCATTAGA

1261 GCTGCTTGCAAACAAAAAAACCACCGCTACCAGCGGTGGTTTGTTTGCCGGATCAAGAGC 1320

CGACGAACGTTTGTTTTTTTGGTGGCGATGGTCGCCACCAAACAAACGGCCTAGTTCTCG

1321 TACCAACTCTTTTTCCGAAGGTAACTGGCTTCAGCAGAGCGCAGATACCAAATACTGTTC 1380

ATGGTTGAGAAAAAGGCTTCCATTGACCGAAGTCGTCTCGCGTCTATGGTTTATGACAAG

1381 TTCTAGTGTAGCCGTAGTTAGGCCACCACTTCAAGAACTCTGTAGCACCGCCTACATACC 1440

AAGATCACATCGGCATCAATCCGGTGGTGAAGTTCTTGAGACATCGTGGCGGATGTATGG

AlwNI

|

1441 TCGCTCTGCTAATCCTGTTACCAGTGGCTGCTGCCAGTGGCGATAAGTCGTGTCTTACCG 1500

AGCGAGACGATTAGGACAATGGTCACCGACGACGGTCACCGCTATTCAGCACAGAATGGC

1501 GGTTGGACTCAAGACGATAGTTACCGGATAAGGCGCAGCGGTCGGGCTGAACGGGGGGTT 1560

CCAACCTGAGTTCTGCTATCAATGGCCTATTCCGCGTCGCCAGCCCGACTTGCCCCCCAA

1561 CGTGCACACAGCCCAGCTTGGAGCGAACGACCTACACCGAACTGAGATACCTACAGCGTG 1620

GCACGTGTGTCGGGTCGAACCTCGCTTGCTGGATGTGGCTTGACTCTATGGATGTCGCAC

1621 AGCTATGAGAAAGCGCCACGCTTCCCGAAGGGAGAAAGGCGGACAGGTATCCGGTAAGCG 1680

TCGATACTCTTTCGCGGTGCGAAGGGCTTCCCTCTTTCCGCCTGTCCATAGGCCATTCGC

1681 GCAGGGTCGGAACAGGAGAGCGCACGAGGGAGCTTCCAGGGGGAAACGCCTGGTATCTTT 1740

CGTCCCAGCCTTGTCCTCTCGCGTGCTCCCTCGAAGGTCCCCCTTTGCGGACCATAGAAA

1741 ATAGTCCTGTCGGGTTTCGCCACCTCTGACTTGAGCGTCGATTTTTGTGATGCTCGTCAG 1800

TATCAGGACAGCCCAAAGCGGTGGAGACTGAACTCGCAGCTAAAAACACTACGAGCAGTC

1801 GGGGGCGGAGCCTATGGAAAAACGCCAGCAACGCGGCCTTTTTACGGTTCCTGGCCTTTT 1860

CCCCCGCCTCGGATACCTTTTTGCGGTCGTTGCGCCGGAAAAATGCCAAGGACCGGAAAA

1861 GCTGGCCTTTTGCTCACATGTTCTTTCCTGCGTTATCCCCTGATTCTGTGGATAACCGTA 1920

CGACCGGAAAACGAGTGTACAAGAAAGGACGCAATAGGGGACTAAGACACCTATTGGCAT

1921 TTACCGCCTTTGAGTGAGCTGATACCGCTCGCCGCAGCCGAACGACCGAGCGCAGCGAGT 1980

AATGGCGGAAACTCACTCGACTATGGCGAGCGGCGTCGGCTTGCTGGCTCGCGTCGCTCA

1981 CAGTGAGCGAGGAAGCGGAAGAGCGCCCAATACGCAAACCGCCTCTCCCCGCGCGTTGGC 2040

GTCACTCGCTCCTTCGCCTTCTCGCGGGTTATGCGTTTGGCGGAGAGGGGCGCGCAACCG

2041 CGATTCATTAATGCAGCTGGCACGACAGGTTTCCCGACTGGAAAGCGGGCAGTGAGCGCA 2100

GCTAAGTAATTACGTCGACCGTGCTGTCCAAAGGGCTGACCTTTCGCCCGTCACTCGCGT

lac prom(2144,2173)>>>

|

2101 ACGCAATTAATGTGAGTTAGCTCACTCATTAGGCACCCCAGGCTTTACACTTTATGCTTC 2160

TGCGTTAATTACACTCAATCGAGTGAGTAATCCGTGGGGTCCGAAATGTGAAATACGAAG

lacZalpha-ccdB_fusion(2218,2871)>>>

|

2161 CGGCTCGTATGTTGTGTGGAATTGTGAGCGGATAACAATTTCACACAGGAAACAGCTATG 2220

M

GCCGAGCATACAACACACCTTAACACTCGCCTATTGTTAAAGTGTGTCCTTTGTCGATAC

SP6 (2236,2259)> HindIII

| |

2221 ACCATGATTACGCCAAGCTATTTAGGTGACACTATAGAATACTCAAGCTATGCATCAAGC 2280

T M I T P S Y L G D T I E Y S S Y A S S

TGGTACTAATGCGGTTCGATAAATCCACTGTGATATCTTATGAGTTCGATACGTAGTTCG

BamHI EcoRI EcoRV

| | |

KpnI SacI SpeI ApoI PstI

| | | | | | |

2281 TTGGTACCGAGCTCGGATCCACTAGTAACGGCCGCCAGTGTGCTGGAATTCTGCAGATAT 2340

L V P S S D P L V T A A S V L E F C R Y

AACCATGGCTCGAGCCTAGGTGATCATTGCCGGCGGTCACACGACCTTAAGACGTCTATA

AvrI

|

AvaI XbaI

| |

NotI XhoI SphI ApaI

| | | | |

2341 CCATCACACTGGCGGCCGCTCGAGCATGCATCTAGAGGGCCCAATTCGCCCTATTCGAAG 2400

P S H W R P L E H A S R G P N S P Y S K

GGTAGTGTGACCGCCGGCGAGCTCGTACGTAGATCTCCCGGGTTAAGCGGGATAAGCTTC

<pZErO_Rs2 (2421,2397)

|

2401 TCGTATTACAATTCACTGGCCGTCGTTTTACAACGTCGTGACTGGGAAAACCCTGGCGTT 2460

S Y Y N S L A V V L Q R R D W E N P G V

AGCATAATGTTAAGTGACCGGCAGCAAAATGTTGCAGCACTGACCCTTTTGGGACCGCAA

2461 ACCCAACTTAATCGCCTTGCAGCACATCCCCCTTTCGCCAGCTGGCGTAATAGCGAAGAG 2520

T Q L N R L A A H P P F A S W R N S E E

TGGGTTGAATTAGCGGAACGTCGTGTAGGGGGAAAGCGGTCGACCGCATTATCGCTTCTC

ccdB (2568,2871)>>>

|

2521 GCCCGCACCGATCGCCCTTCCCAACAGTTGCGCAGCCTATACGTACGGCAGTTTAAGGTT 2580

A R T D R P S Q Q L R S L Y V R Q F K V

CGGGCGTGGCTAGCGGGAAGGGTTGTCAACGCGTCGGATATGCATGCCGTCAAATTCCAA

2581 TACACCTATAAAAGAGAGAGCCGTTATCGTCTGTTTGTGGATGTACAGAGTGATATTATT 2640

Y T Y K R E S R Y R L F V D V Q S D I I

ATGTGGATATTTTCTCTCTCGGCAATAGCAGACAAACACCTACATGTCTCACTATAATAA

2641 GACACGCCGGGGCGACGGATGGTGATCCCCCTGGCCAGTGCACGTCTGCTGTCAGATAAA 2700

D T P G R R M V I P L A S A R L L S D K

CTGTGCGGCCCCGCTGCCTACCACTAGGGGGACCGGTCACGTGCAGACGACAGTCTATTT

2701 GTCTCCCGTGAACTTTACCCGGTGGTGCATATCGGGGATGAAAGCTGGCGCATGATGACC 2760

V S R E L Y P V V H I G D E S W R M M T

CAGAGGGCACTTGAAATGGGCCACCACGTATAGCCCCTACTTTCGACCGCGTACTACTGG

2761 ACCGATATGGCCAGTGTGCCGGTCTCCGTTATCGGGGAAGAAGTGGCTGATCTCAGCCAC 2820

T D M A S V P V S V I G E E V A D L S H

TGGCTATACCGGTCACACGGCCAGAGGCAATAGCCCCTTCTTCACCGACTAGAGTCGGTG

2821 CGCGAAAATGACATCAAAAACGCCATTAACCTGATGTTCTGGGGAATATAACTTGGCACT 2880

R E N D I K N A I N L M F W G I *

GCGCTTTTACTGTAGTTTTTGCGGTAATTGGACTACAAGACCCCTTATATTGAACCGTGA

2881 GGCCGTCGTTTTACAACGTCGTGACTGGGAAAACCCTGGCGTTACCCAACTTAATCGCCT 2940

CCGGCAGCAAAATGTTGCAGCACTGACCCTTTTGGGACCGCAATGGGTTGAATTAGCGGA

2941 TGCAGCACATCCCCCTTTCGCCAGCTGGCGTAATAGCGAAGAGGCCCGCACCGATCGCCC 3000

ACGTCGTGTAGGGGGAAAGCGGTCGACCGCATTATCGCTTCTCCGGGCGTGGCTAGCGGG

NarI

|

3001 TTCCCAACAGTTGCGCAGCCTGAATGGCGAATGGCGCCTGATGCGGTATTTTCTCCTTAC 3060

AAGGGTTGTCAACGCGTCGGACTTACCGCTTACCGCGGACTACGCCATAAAAGAGGAATG

NdeI

|

3061 GCATCTGTGCGGTATTTCACACCGCATATGGTGCACTCTCAGTACAATCTGCTCTGATGC 3120

CGTAGACACGCCATAAAGTGTGGCGTATACCACGTGAGAGTCATGTTAGACGAGACTACG

3121 CGCATAGTTAAGCCAGCCCCGACACCCGCCAACACCCGCTGACGCGCCCTGACGGGCTTG 3180

GCGTATCAATTCGGTCGGGGCTGTGGGCGGTTGTGGGCGACTGCGCGGGACTGCCCGAAC

3181 TCTGCTCCCGGCATCCGCTTACAGACAAGCTGTGACCGTCTCCGGGAGCTGCATGTGTCA 3240

AGACGAGGGCCGTAGGCGAATGTCTGTTCGACACTGGCAGAGGCCCTCGACGTACACAGT

3241 GAGGTTTTCACCGTCATCACCGAAACGCGCGA 3272

CTCCAAAAGTGGCAGTAGTGGCTTTGCGCGCT
