# Supplementary material for: A novel series of high-efficiency vectors for TA cloning and blunt-end cloning of PCR products
Source: Sci Rep. 2019 Apr 23;9:6417. doi: 10.1038/s41598-019-42868-6 (PMC6478821; doi:10.1038/s41598-019-42868-6)
Supplement: Supplementary file 2 — Dataset S1 [file 41598_2019_42868_MOESM2_ESM.zip › Supplementary_DatasetS1/pCRZeroT_txt_map.docx]

>pCRZeroT_2975bp

1 GACGAAAGGGCCTCGTGATACGCCTATTTTTATAGGTTAATGTCATGATAATAATGGTTT 60

CTGCTTTCCCGGAGCACTATGCGGATAAAAATATCCAATTACAGTACTATTATTACCAAA

61 CTTAGACGTCAGGTGGCACTTTTCGGGGAAATGTGCGCGGAACCCCTATTTGTTTATTTT 120

GAATCTGCAGTCCACCGTGAAAAGCCCCTTTACACGCGCCTTGGGGATAAACAAATAAAA

amp prom(131,159)>>>

|

121 TCTAAATACATTCAAATATGTATCCGCTCATGAGACAATAACCCTGATAAATGCTTCAAT 180

AGATTTATGTAAGTTTATACATAGGCGAGTACTCTGTTATTGGGACTATTTACGAAGTTA

amp marker(201,1061)>>>

|

181 AATATTGAAAAAGGAAGAGTATGAGTATTCAACATTTCCGTGTCGCCCTTATTCCCTTTT 240

TTATAACTTTTTCCTTCTCATACTCATAAGTTGTAAAGGCACAGCGGGAATAAGGGAAAA

241 TTGCGGCATTTTGCCTTCCTGTTTTTGCTCACCCAGAAACGCTGGTGAAAGTAAAAGATG 300

AACGCCGTAAAACGGAAGGACAAAAACGAGTGGGTCTTTGCGACCACTTTCATTTTCTAC

301 CTGAAGATCAGTTGGGTGCACGAGTGGGTTACATCGAACTGGATCTCAACAGCGGTAAGA 360

GACTTCTAGTCAACCCACGTGCTCACCCAATGTAGCTTGACCTAGAGTTGTCGCCATTCT

361 TCCTTGAGAGTTTTCGCCCCGAAGAACGTTTTCCAATGATGAGCACTTTTAAAGTTCTGC 420

AGGAACTCTCAAAAGCGGGGCTTCTTGCAAAAGGTTACTACTCGTGAAAATTTCAAGACG

421 TATGTGGCGCGGTATTATCCCGTATTGACGCCGGGCAAGAGCAACTCGGTCGCCGCATAC 480

ATACACCGCGCCATAATAGGGCATAACTGCGGCCCGTTCTCGTTGAGCCAGCGGCGTATG

481 ACTATTCTCAGAATGACTTGGTTGAGTACTCACCAGTCACAGAAAAGCATCTTACGGATG 540

TGATAAGAGTCTTACTGAACCAACTCATGAGTGGTCAGTGTCTTTTCGTAGAATGCCTAC

541 GCATGACAGTAAGAGAATTATGCAGTGCTGCCATAACCATGAGTGATAACACTGCGGCCA 600

CGTACTGTCATTCTCTTAATACGTCACGACGGTATTGGTACTCACTATTGTGACGCCGGT

601 ACTTACTTCTGACAACGATCGGAGGACCGAAGGAGCTAACCGCTTTTTTGCACAACATGG 660

TGAATGAAGACTGTTGCTAGCCTCCTGGCTTCCTCGATTGGCGAAAAAACGTGTTGTACC

661 GGGATCATGTAACTCGCCTTGATCGTTGGGAACCGGAGCTGAATGAAGCCATACCAAACG 720

CCCTAGTACATTGAGCGGAACTAGCAACCCTTGGCCTCGACTTACTTCGGTATGGTTTGC

721 ACGAGCGTGACACCACGATGCCTGTAGCAATGGCAACAACGTTGCGCAAACTATTAACTG 780

TGCTCGCACTGTGGTGCTACGGACATCGTTACCGTTGTTGCAACGCGTTTGATAATTGAC

781 GCGAACTACTTACTCTAGCTTCCCGGCAACAATTAATAGACTGGATGGAGGCGGATAAAG 840

CGCTTGATGAATGAGATCGAAGGGCCGTTGTTAATTATCTGACCTACCTCCGCCTATTTC

841 TTGCAGGACCACTTCTGCGCTCGGCCCTTCCGGCTGGCTGGTTTATTGCTGATAAATCTG 900

AACGTCCTGGTGAAGACGCGAGCCGGGAAGGCCGACCGACCAAATAACGACTATTTAGAC

901 GAGCCGGTGAGCGTGGGTCTCGCGGTATCATTGCAGCACTGGGGCCAGATGGTAAGCCCT 960

CTCGGCCACTCGCACCCAGAGCGCCATAGTAACGTCGTGACCCCGGTCTACCATTCGGGA

961 CCCGTATCGTAGTTATCTACACGACGGGGAGTCAGGCAACTATGGATGAACGAAATAGAC 1020

GGGCATAGCATCAATAGATGTGCTGCCCCTCAGTCCGTTGATACCTACTTGCTTTATCTG

1021 AGATCGCTGAGATAGGTGCCTCACTGATTAAGCATTGGTAACTGTCAGACCAAGTTTACT 1080

TCTAGCGACTCTATCCACGGAGTGACTAATTCGTAACCATTGACAGTCTGGTTCAAATGA

1081 CATATATACTTTAGATTGATTTAAAACTTCATTTTTAATTTAAAAGGATCTAGGTGAAGA 1140

GTATATATGAAATCTAACTAAATTTTGAAGTAAAAATTAAATTTTCCTAGATCCACTTCT

1141 TCCTTTTTGATAATCTCATGACCAAAATCCCTTAACGTGAGTTTTCGTTCCACTGAGCGT 1200

AGGAAAAACTATTAGAGTACTGGTTTTAGGGAATTGCACTCAAAAGCAAGGTGACTCGCA

pMB1 origin(1216,1835)>>>

|

1201 CAGACCCCGTAGAAAAGATCAAAGGATCTTCTTGAGATCCTTTTTTTCTGCGCGTAATCT 1260

GTCTGGGGCATCTTTTCTAGTTTCCTAGAAGAACTCTAGGAAAAAAAGACGCGCATTAGA

1261 GCTGCTTGCAAACAAAAAAACCACCGCTACCAGCGGTGGTTTGTTTGCCGGATCAAGAGC 1320

CGACGAACGTTTGTTTTTTTGGTGGCGATGGTCGCCACCAAACAAACGGCCTAGTTCTCG

1321 TACCAACTCTTTTTCCGAAGGTAACTGGCTTCAGCAGAGCGCAGATACCAAATACTGTTC 1380

ATGGTTGAGAAAAAGGCTTCCATTGACCGAAGTCGTCTCGCGTCTATGGTTTATGACAAG

1381 TTCTAGTGTAGCCGTAGTTAGGCCACCACTTCAAGAACTCTGTAGCACCGCCTACATACC 1440

AAGATCACATCGGCATCAATCCGGTGGTGAAGTTCTTGAGACATCGTGGCGGATGTATGG

AlwNI

|

1441 TCGCTCTGCTAATCCTGTTACCAGTGGCTGCTGCCAGTGGCGATAAGTCGTGTCTTACCG 1500

AGCGAGACGATTAGGACAATGGTCACCGACGACGGTCACCGCTATTCAGCACAGAATGGC

1501 GGTTGGACTCAAGACGATAGTTACCGGATAAGGCGCAGCGGTCGGGCTGAACGGGGGGTT 1560

CCAACCTGAGTTCTGCTATCAATGGCCTATTCCGCGTCGCCAGCCCGACTTGCCCCCCAA

1561 CGTGCACACAGCCCAGCTTGGAGCGAACGACCTACACCGAACTGAGATACCTACAGCGTG 1620

GCACGTGTGTCGGGTCGAACCTCGCTTGCTGGATGTGGCTTGACTCTATGGATGTCGCAC

1621 AGCTATGAGAAAGCGCCACGCTTCCCGAAGGGAGAAAGGCGGACAGGTATCCGGTAAGCG 1680

TCGATACTCTTTCGCGGTGCGAAGGGCTTCCCTCTTTCCGCCTGTCCATAGGCCATTCGC

1681 GCAGGGTCGGAACAGGAGAGCGCACGAGGGAGCTTCCAGGGGGAAACGCCTGGTATCTTT 1740

CGTCCCAGCCTTGTCCTCTCGCGTGCTCCCTCGAAGGTCCCCCTTTGCGGACCATAGAAA

1741 ATAGTCCTGTCGGGTTTCGCCACCTCTGACTTGAGCGTCGATTTTTGTGATGCTCGTCAG 1800

TATCAGGACAGCCCAAAGCGGTGGAGACTGAACTCGCAGCTAAAAACACTACGAGCAGTC

1801 GGGGGCGGAGCCTATGGAAAAACGCCAGCAACGCGGCCTTTTTACGGTTCCTGGCCTTTT 1860

CCCCCGCCTCGGATACCTTTTTGCGGTCGTTGCGCCGGAAAAATGCCAAGGACCGGAAAA

1861 GCTGGCCTTTTGCTCACATGTTCTTTCCTGCGTTATCCCCTGATTCTGTGGATAACCGTA 1920

CGACCGGAAAACGAGTGTACAAGAAAGGACGCAATAGGGGACTAAGACACCTATTGGCAT

1921 TTACCGCCTTTGAGTGAGCTGATACCGCTCGCCGCAGCCGAACGACCGAGCGCAGCGAGT 1980

AATGGCGGAAACTCACTCGACTATGGCGAGCGGCGTCGGCTTGCTGGCTCGCGTCGCTCA

1981 CAGTGAGCGAGGAAGCGGAAGAGCGCCCAATACGCAAACCGCCTCTCCCCGCGCGTTGGC 2040

GTCACTCGCTCCTTCGCCTTCTCGCGGGTTATGCGTTTGGCGGAGAGGGGCGCGCAACCG

2041 CGATTCATTAATGCAGCTGGCACGACAGGTTTCCCGACTGGAAAGCGGGCAGTGAGCGCA 2100

GCTAAGTAATTACGTCGACCGTGCTGTCCAAAGGGCTGACCTTTCGCCCGTCACTCGCGT

pUC18-118F (2146,2169)>

|

lac prom(2144,2173)>>>

| |

2101 ACGCAATTAATGTGAGTTAGCTCACTCATTAGGCACCCCAGGCTTTACACTTTATGCTTC 2160

TGCGTTAATTACACTCAATCGAGTGAGTAATCCGTGGGGTCCGAAATGTGAAATACGAAG

M13F(2186,2209)> lacZalpha-ccdB_fusion(2218,2553)>>>

| |

2161 CGGCTCGTATGTTGTGTGGAATTGTGAGCGGATAACAATTTCACACAGGAAACAGCTATG 2220

M

GCCGAGCATACAACACACCTTAACACTCGCCTATTGTTAAAGTGTGTCCTTTGTCGATAC

EcoRI 　　　　　　　pCRZeroTccdF (2254,2277)>

|　　　　　　　　　　|

ApoI XcmI(2) ccdB(2254,2556)>>>

| | |

2221 ACCATGATTACGAATTCGCCAatacttgtaTGGcagtttaaggtttacacctataaaaga 2280

T M I T N S P I L V W Q F K V Y T Y K R

TGGTACTAATGCTTAAGCGGTTATGAACATACCGTCAAATTCCAAATGTGGATATTTTCT

SmaI

|

AvaI

| |

AvrI

| |

XmaI

| |

2281 gagagccgttatcgtctgtttgtggatgtacagagtgatattattgacacgcccgggcga 2340

E S R Y R L F V D V Q S D I I D T P G R

CTCTCGGCAATAGCAGACAAACACCTACATGTCTCACTATAATAACTGTGCGGGCCCGCT

2341 cggatggtgatccccctggccagtgcacgtctgctgtcagataaagtctcccgtgaactt 2400

R M V I P L A S A R L L S D K V S R E L

GCCTACCACTAGGGGGACCGGTCACGTGCAGACGACAGTCTATTTCAGAGGGCACTTGAA

<pCRZeroTccdR (2426,2403)

|

2401 tacccggtggtgcatatcggggatgaaagctggcgcatgatgaccaccgatatggccagt 2460

Y P V V H I G D E S W R M M T T D M A S

ATGGGCCACCACGTATAGCCCCTACTTTCGACCGCGTACTACTGGTGGCTATACCGGTCA

2461 gtgccggtctccgttatcggggaagaagtggctgatctcagccaccgcgaaaatgacatc 2520

V P V S V I G E E V A D L S H R E N D I

CACGGCCAGAGGCAATAGCCCCTTCTTCACCGACTAGAGTCGGTGGCGCTTTTACTGTAG

lacZalpha(2573,2830)>>>

XcmI(2) |HindIII

| ||

2521 aaaaacgccattaacctgatgttctggggaatataaCCAtacaagtatTGGAAGCTTGGC 2580

K N A I N L M F W G I * S L A

TTTTTGCGGTAATTGGACTACAAGACCCCTTATATTGGTATGTTCATAACCTTCGAACCG

<M13R(2601,2624)

|

2581 ACTGGCCGTCGTTTTACAACGTCGTGACTGGGAAAACCCTGGCGTTACCCAACTTAATCG 2640

L A V V L Q R R D W E N P G V T Q L N R

TGACCGGCAGCAAAATGTTGCAGCACTGACCCTTTTGGGACCGCAATGGGTTGAATTAGC

< pUC18-118R (2662,2639)

|

2641 CCTTGCAGCACATCCCCCTTTCGCCAGCTGGCGTAATAGCGAAGAGGCCCGCACCGATCG 2700

L A A H P P F A S W R N S E E A R T D R

GGAACGTCGTGTAGGGGGAAAGCGGTCGACCGCATTATCGCTTCTCCGGGCGTGGCTAGC

NarI

|

2701 CCCTTCCCAACAGTTGCGCAGCCTGAATGGCGAATGGCGCCTGATGCGGTATTTTCTCCT 2760

P S Q Q L R S L N G E W R L M R Y F L L

GGGAAGGGTTGTCAACGCGTCGGACTTACCGCTTACCGCGGACTACGCCATAAAAGAGGA

NdeI

|

2761 TACGCATCTGTGCGGTATTTCACACCGCATATGGTGCACTCTCAGTACAATCTGCTCTGA 2820

T H L C G I S H R I W C T L S T I C S D

ATGCGTAGACACGCCATAAAGTGTGGCGTATACCACGTGAGAGTCATGTTAGACGAGACT

2821 TGCCGCATAGTTAAGCCAGCCCCGACACCCGCCAACACCCGCTGACGCGCCCTGACGGGC 2880

A A *

ACGGCGTATCAATTCGGTCGGGGCTGTGGGCGGTTGTGGGCGACTGCGCGGGACTGCCCG

2881 TTGTCTGCTCCCGGCATCCGCTTACAGACAAGCTGTGACCGTCTCCGGGAGCTGCATGTG 2940

AACAGACGAGGGCCGTAGGCGAATGTCTGTTCGACACTGGCAGAGGCCCTCGACGTACAC

2941 TCAGAGGTTTTCACCGTCATCACCGAAACGCGCGA 2975

AGTCTCCAAAAGTGGCAGTAGTGGCTTTGCGCGCT
